# Experimental Demonstration of Correlation-Assisted Energy Extraction Gain

**DOI:** 10.3390/e28101079

**Published:** 2026-09-30

**Authors:** Alexander Stahl, Michael Kewming, John Goold, Janine Hilder, Ulrich Georg Poschinger, Ferdinand Schmidt-Kaler

**Affiliations:** 1 QUANTUM, Institut für Physik, Universität Mainz, Staudingerweg 7, 55128 Mainz, Germany; 2 School of Physics, Trinity College Dublin, College Green, Dublin, Ireland

**Keywords:** quantum thermodynamics, Maxwell’s demon, quantum correlations, trapped ions, feedback control, energy extraction

## Abstract

Within the framework of microscopic thermodynamics, correlations can play a crucial role in energy extraction. We experimentally investigated the link between information encoded in tunable correlations and its utility for feedback-assisted energy transfer. We realized an agent-demon protocol involving two trapped-ion qubits and show that an appropriate feedback policy increases the average energy stored in the demon qubit by capitalizing on correlations between the constituents. For the chosen family of nearly pure resource states, we quantify both the concurrence and the feedback-assisted energy gain and observe the predicted relation between them. We also show theoretically that the same fixed protocol yields the same gain after complete dephasing in the local energy basis. The experiment establishes a concurrence–gain relation within the investigated pure-state family.

## 1. Introduction

Microscopic thermodynamics is a prospering field in modern physics, studying how concepts and notions from classical thermodynamics carry over to the microscopic domain, where fluctuations of a thermal or quantum nature play a significant role. By adapting classical principles of thermodynamics to microscopic systems, researchers have gained invaluable insights into the resources that underpin the operation of microscopic engines and other quantum machines [1,2,3,4,5]. This has lead to the identification of specific thermodynamic properties of quantum thermal machines [6,7,8,9,10,11,12,13,14], which operate by manipulating the quantum state of a system to extract energy or perform work [15]. Technological advances within the last two decades have enabled experimenters to extract information from a microscopic system and conditionally act in response to it, thus bringing the concept of a *demon* to reality [16,17]. Generalizing the famous Maxwell demon, this notion describes an unsupervised entity acting within a physical system by acquiring information and acting in response to it. Studying the impact of such demons on the properties of physical systems is an active field of research [18,19].

However, despite the growing body of research on the thermodynamic features of quantum systems, a contentious debate surrounds the specific role of correlations, particularly non-classical correlations, in energy extraction schemes [20,21,22,23]. The identification of an intricate link between thermodynamics and entanglement [24] has sparked intense interest in understanding the role of non-classical correlations as resources for thermodynamic processes [25,26]. As highlighted in [27], non-classical correlations provide a lower bound on the extractable energy, but classically correlated states can outperform them, producing higher yields.

Here, we present an experimental demonstration of a minimal protocol for correlation-assisted energy transfer in a bipartite system. It is realized by employing qubits encoded in trapped atomic ions. The strengths of this versatile platform—particularly the availability of precise methods for manipulating and reading out qubits—render it ideally suited for applications in microscopic and quantum thermodynamics [28,29,30,31]. The protocol is based on two parties, a *demon* and an *agent*, each associated with one constituent of a two-qubit state, henceforth referred to as the *resource*. These names denote operationally asymmetric roles rather than the interchangeable labels Alice and Bob: in analogy with Maxwell’s demon, the demon obtains information by measuring her qubit and uses the outcome to choose a global feedback operation, whereas the agent labels the other qubit whose excitation can be transferred. The demon’s objective is to increase the energy stored in her qubit by minimizing the probability of the agent’s qubit concluding in the excited state. While the demon possesses knowledge about the initial resource state and the capability to implement joint unitary operations on the system, she lacks the ability to measure the state of the agent’s qubit. The demon uses a measurement in conjunction with knowledge about correlations to acquire information regarding the state of the agent. Subsequently, she utilizes the acquired information to apply feedback [27,32]. The quantity of interest is the feedback-assisted gain δW, i.e., the increase in the average energy stored in the demon qubit relative to the case without feedback.

Reference [27] considers demonic ergotropy: a projective measurement on an ancilla is followed by an outcome-dependent cyclic unitary on the system, optimized to maximize extractable work. In this framework, for pure resource states, the optimized gain is related to entanglement, and for two qubits, a concurrence-dependent lower bound is (The additional factor 1/2 does not appear in the bound given in Ref. [27], as the free Hamiltonian is different (${\hat{H}}_{0}=-{\hat{\sigma }}_{z}$)) as follows:

(1) $$\delta W\geq \frac{1}{2}\left(1-\sqrt{1-C^{2}}\right)$$

In this experiment, we instead implement a fixed joint conditional-SWAP feedback operation and measure the resulting local energy increase for the demon qubit. Equation (1) is therefore included as a benchmark from the demonic ergotropy framework, not as a bound derived for our fixed protocol.

## 2. Protocol for Correlation-Assisted Energy Transfer

**Figure 1:**
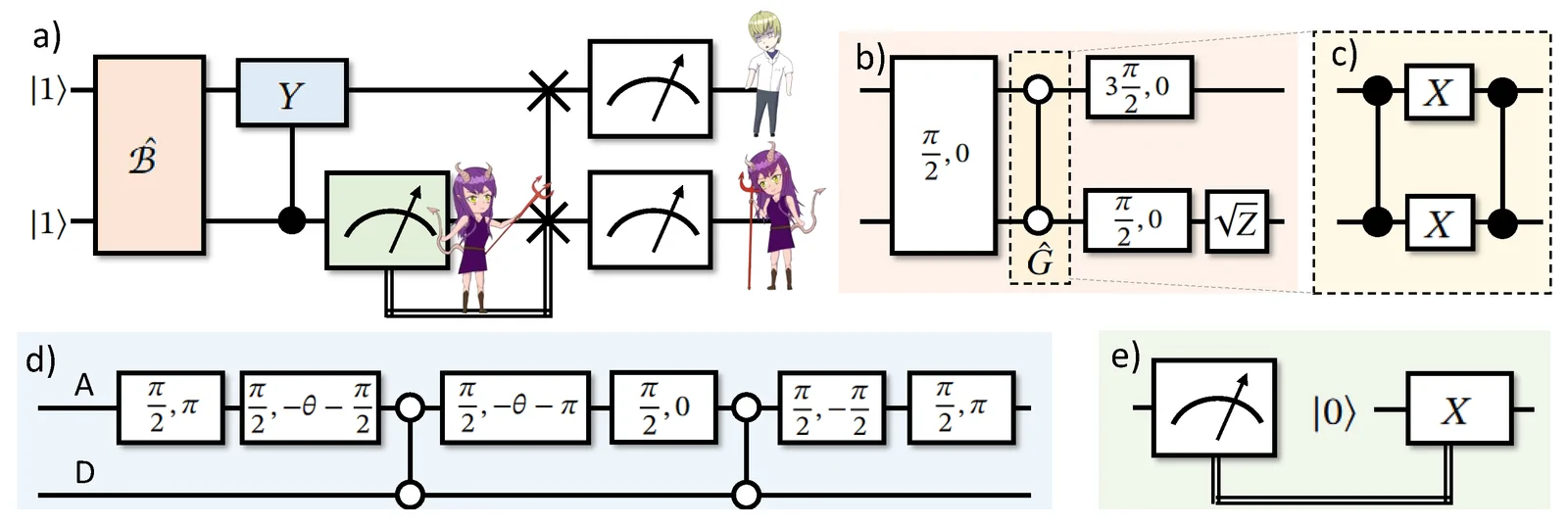
(**a**) Quantum circuit for the correlation-enabled work extraction protocol. Both qubits are prepared in a Bell state via a gate sequence $\hat{B}$, for which the decomposition into native gates is shown in (**b**). The further decomposition of the entangling gate $\hat{G}$, including rephasing pulses and indicated with open circles, is shown in (**c**), where the gates indicated with filled circles are π/4 phase gates (see text). The decomposition of the parametric controlled-*Y* gate used to prepare the state |Ψ(θ)〉 is shown in (**d**) and explained in the Supplementary Materials. The emulation of the demon’s QND measurement is shown in (**e**). The demon performs a SWAP operation conditioned on the measurement outcome.

The work extraction protocol is described by a quantum circuit operating on two qubits, shown in Figure 1. Both qubits are described by a free Hamiltonian ${\hat{H}}_{0}=\hbar \omega |1\rangle \langle 1|$, where |1〉 corresponds to the energetically higher state and ℏω=ϵ is the qubit energy splitting. The initial resource state provided to the demon and agent is

(2) $$|\Psi (\theta )\rangle =\frac{1}{\sqrt{2}}\left(\cos(\theta )\;|1_{A}0_{D}\rangle +|0_{A}1_{D}\rangle -\sin(\theta )\;|0_{A}0_{D}\rangle \right)$$

This state is obtained by preparing a maximally entangled Bell state $|\Psi^{+}\rangle =\left(|0_{A}1_{D}\rangle +|1_{A}0_{D}\rangle \right)/\sqrt{2}$ and applying a parametric controlled-*Y* gate $c\hat{Y}\left(\theta \right)=1_{A}⊗|1_{D}\rangle \langle 1_{D}|+e^{-i\frac{\theta }{2}{\hat{Y}}_{A}}⊗|0_{D}\rangle \langle 0_{D}|$. The angle θ controls the number of correlations: for θ=0, the maximally entangled Bell state is retained, while for θ=π/2, the agent’s and demon’s qubits are in separable states. The entanglement between the agent’s and the demon’s qubits can be quantified via the concurrence C [33]

(3) $$\begin{array}{c} C=\max\{0,\lambda_{1}-\lambda_{2}-\lambda_{3}-\lambda_{4}\}\;, \end{array}$$

where $\lambda_{i}$ represents the eigenvalues of $R=\sqrt{\sqrt{\rho }\tilde{\rho }\sqrt{\rho }}$, with $\tilde{\rho }=\left(\sigma_{y}⊗\sigma_{y}\right)\rho^{*}\left(\sigma_{y}⊗\sigma_{y}\right)$, and $\rho^{*}$ is the conjugate of ρ. For the initial state Equation (2), this yields

(4) C(θ)=|cosθ|

Indeed, for a pure two-qubit state with a coefficient matrix *M*, the concurrence is C=2|detM|. Here, $M=\frac{1}{\sqrt{2}}\left(\begin{array}{cc} -\sin\theta & 1\\ \cos\theta & 0 \end{array}\right)$, directly yielding Equation (4). With a resource state Equation (2) prepared, the protocol is as follows: The demon performs a projective measurement on her qubit in the energy basis, yielding outcomes $d=0_{D}$ or $d=1_{D}$ at balanced probabilities $Pr\left(d=0_{D}\right)=Pr\left(d=1_{D}\right)=1/2$. She uses this information to execute a feedback mechanism, consisting of the application of a SWAP gate between both qubits (${\hat{U}}_{0}$), which is only carried out once measurement result $0_{D}$ has been obtained; otherwise, no operation is carried out (${\hat{U}}_{1}=1$). This policy is optimal, as swapping the qubits is the best course of action for the demon, upon having obtained measurement result $0_{D}$. With the resource state $\hat{\rho }=|\Psi (\theta )\rangle \langle \Psi (\theta )|$, the projection operators ${\hat{\Pi }}_{d}=1_{A}⊗|d\rangle \langle d|$, and the post-measurement states

(5) $${\hat{\rho }}_{d}={\hat{\Pi }}_{d}\;\hat{\rho }\;{\hat{\Pi }}_{d}/\mathrm{Pr}\left(d\right),$$

the final state after execution of the feedback operation is given by

(6) $$\begin{array}{ccc} {\hat{\rho }}^{\prime } & = & \frac{1}{2}{\hat{U}}_{0}{\hat{\rho }}_{0_{D}}{\hat{U}}_{0}^{†}+\frac{1}{2}{\hat{U}}_{1}{\hat{\rho }}_{1_{D}}{\hat{U}}_{1}^{†}. \end{array}$$

The feedback-assisted gain is given by the increase in the demon’s local average energy after applying the conditional-SWAP policy:

(7) $$\delta W=\mathrm{Tr}\left[{\hat{H}}_{0,D}\left({\hat{\rho }}^{\prime }-\hat{\rho }\right)\right]$$

Evaluating Equation (7) for the state family in Equation (2) reveals the following relation between the gain and the concurrence (see the Supplemental Materials):

(8) $$\frac{\delta W}{\epsilon }=\frac{C{\left(\theta \right)}^{2}}{2}.$$

For the present measurement and conditional-SWAP protocol, the operational origin of this result is particularly transparent. For an arbitrary input state $\hat{\rho }$, the gain is

(9) $$\frac{\delta W}{\epsilon }=\langle 1_{A}0_{D}|\hat{\rho }|1_{A}0_{D}\rangle ,$$

because it is precisely this population that is transferred from the agent excitation to the demon excitation. Considering the initial states Equation (2) after complete dephasing in the local energy basis gives states which are separable but have the same local populations, total average energy, and gain $\delta W/\epsilon =\cos^{2}\theta /2$. Thus, Equation (8) is valid within the selected pure-state family, in which concurrence and energy populations vary together; it does not establish a general relation between entanglement and the observed gain.

## 3. Experimental Realization

The qubit platform employed for this work is atomic ^40^${Ca}^{+}$ ions stored in a segmented Paul trap [34]. The qubits are encoded in the spin of the ion’s valence electron [31,35] (See also the Supplemental Materials). The qubit states pertain to the spin’s alignment with an external magnetic field, yielding an energy splitting of ϵ/ℏ≈2π×10 MHz. The qubits are initialized via optical pumping and read out via population transfer from state |0〉 to the metastable $D_{5/2}$ state, while detection relies on recording state-dependent resonance fluorescence [35]. All laser-driven operations take place at a fixed trap site, the laser interaction zone (LIZ). The ions are shuttled within the segmented trap by supplying suitable electric waveforms to the trap electrodes [36] such that either only the demon’s qubit, only the agent’s qubit, or both at the same time can undergo laser-driven operations or provide a qubit readout. Qubit rotations of the form $\hat{R}\left(\theta ,\phi \right)=\exp\left[-\frac{i}{2}\theta \left(\cos\phi \hat{X}+\sin\phi \hat{Y}\right)\right]$, including Pauli gates, are driven by a pair of co-propagating far-off-resonant laser beams generating resonant stimulated Raman transitions. Two-qubit π/4-phase gates of the form ${\hat{U}}_{ZZ}=\exp\left[i\frac{\pi }{8}\hat{Z}⊗\hat{Z}\right]$ are driven by a pair of counter-propagating far-off-resonant laser beams [37,38]. These are combined with qubit rephasing rotations to generate a maximally entangling gate $\hat{G}={\hat{U}}_{ZZ}\hat{R}\left(\pi ,0\right){\hat{U}}_{ZZ}$, as shown in Figure 1c. The initial odd-parity Bell state $|\Psi^{+}\rangle$ is prepared by simultaneously preparing both qubits in superposition states, acting with two ${\hat{U}}_{ZZ}$ gates interspersed with simultaneous Pauli-*X* gates for suppressing gate errors [39]; see Figure 1b. The preparation of the resource state is concluded by a controlled-*Y* gate with a variable rotation angle, which is decomposed in two ${\hat{U}}_{ZZ}$ gates and local rotations, as shown in Figure 1d.

The protocol requires real-time feedback operations, conditioned on mid-sequence measurements. These operations are carried out by (i) accumulating photon counts on a system-on-a-chip (SoC) device, (ii) comparing the count number to a specified threshold on the SoC to discriminate measurement results ‘0’ (a count below the threshold) and ‘1’ (a count above the threshold), and (iii) employing a real-time logic in the SoC that decides which stored voltage ramps are to be generated and sent to the trap electrodes. 

As the demon’s first qubit measurement via state-dependent fluorescence is completely depolarizing, an ideal QND measurement has to be realized via feedback, as shown in Figure 1e: After the measurement, the demon’s qubit is re-initialized to $|0_{D}\rangle$ via optical pumping. If the qubit was previously detected in $|1_{D}\rangle$, it is kept in the LIZ; otherwise it is moved away. In any case, laser pulses for a Pauli-*X* gate are emitted such that the state is only flipped to $|1_{D}\rangle$ for previous detection in $|1_{D}\rangle$. The conditional SWAP gate enacted by the demon upon detecting her qubit in $|0_{D}\rangle$ is driven by voltage ramps that rotate the two-ion Coulomb crystal such that their identities are swapped [40]. This SWAP is carried out only when the demon’s qubit has previously been measured in $|0_{D}\rangle$.

## 4. Results

**Figure 2:**
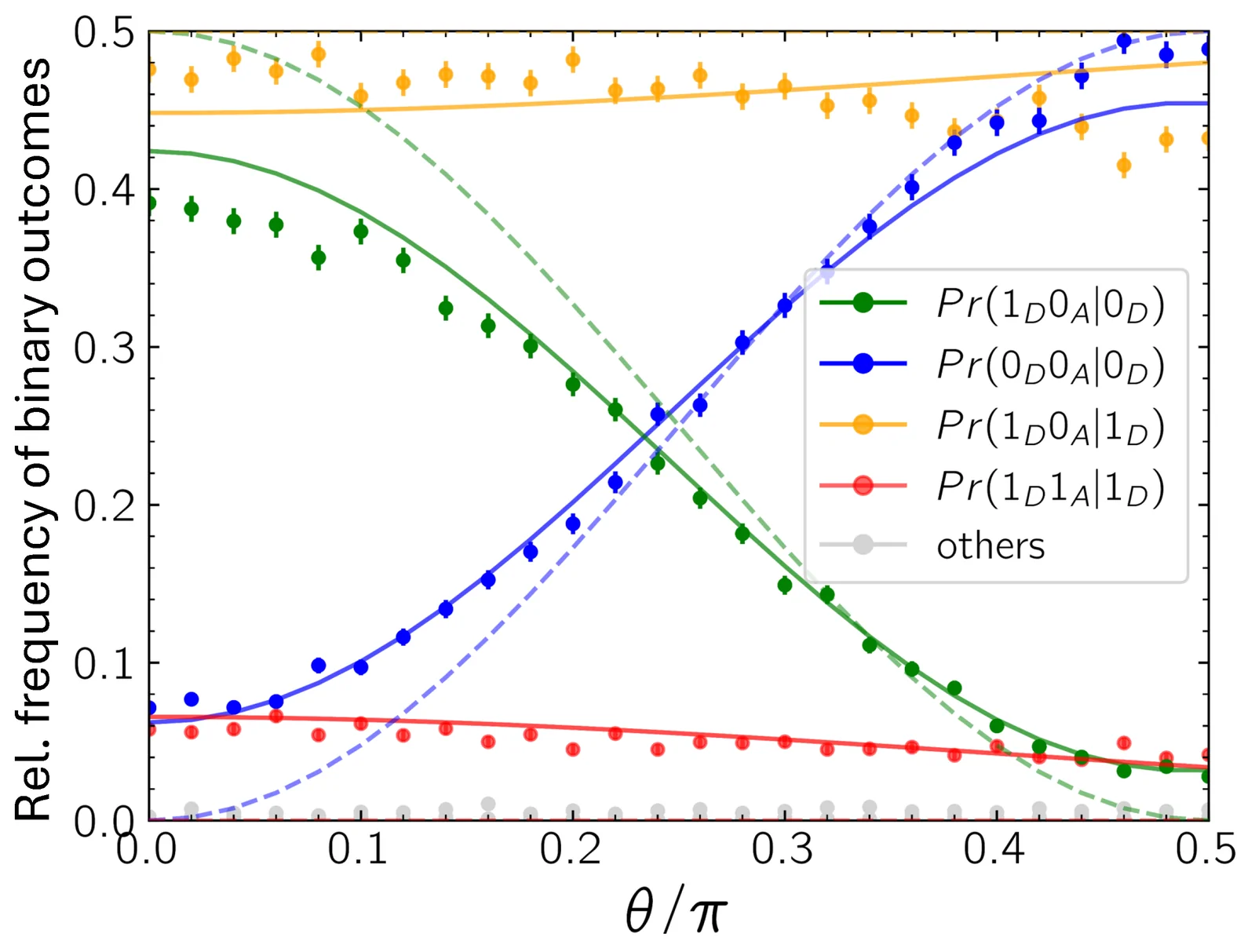
Measured relative frequencies of binary outcomes $\mathrm{Pr}(d^{\prime }a^{\prime }|d)$ versus the preparation angle θ. The probability that the demon will gain an excitation after swapping her qubit with the agent’s one (green) decreases with θ, while the probability of energy extraction by neither party (blue) increases with θ. The probability of an initial demon excitation and no swap operation (yellow) remains constant, and simultaneous final excitations of both parties (red) are due to gate errors (see text). Further outcomes (cumulated, grey) are due to preparation and measurement errors. Each data point corresponds to 3500 independent and identical repetitions of the protocol. Dashed lines show the ideal predictions; solid lines show the circuit simulation with the three controlled-phase deviations fitted to these population data, as described in the text.

The protocol was executed with the preparation angle θ varying between 0 and π/2. For each value of θ, independent repetitions of the protocol were carried out. We estimated the joint probabilities for each final agent and demon outcomes $\mathrm{Pr}(d^{\prime },a^{\prime }|d)$, conditioned on the demon’s mid-circuit outcome *d*, using the relative frequencies for each outcome. The measured outcome probabilities versus θ are shown in Figure 2. The probability $\mathrm{Pr}(1_{D}0_{A}|0_{D})$ that the demon will gain an excitation by swapping the qubits decreases with the preparation angle θ or, equivalently, with decreasing correlations of the resource. The demon’s final average energy is obtained from the marginal probability of measurement outcome $d_{D}^{\prime }=1_{D}$:

(10) $$\frac{E_{f}}{\epsilon }=\mathrm{Pr}\left(d_{D}^{\prime }=1_{D}\right).$$

Likewise, the demon’s average energy before applying feedback is determined by the first measurement outcome:

(11) $$\frac{E_{i}}{\epsilon }=\mathrm{Pr}\left(d_{D}=1_{D}\right).$$

$E_{i}$ and $E_{f}$ are directly measured local average energies, and $\delta W=E_{f}-E_{i}$ gives the feedback-assisted gain according to Equation (7). Interpreting this quantity as a gain of extractable work assumes there is a subsequent controlled energy transfer from qubit *D* to an ideal work-storage degree of freedom. This operational quantity is distinct from the ergotropy of the unconditioned reduced density matrix and from the optimized demonic ergotropy of Ref. [27].

**Figure 3:**
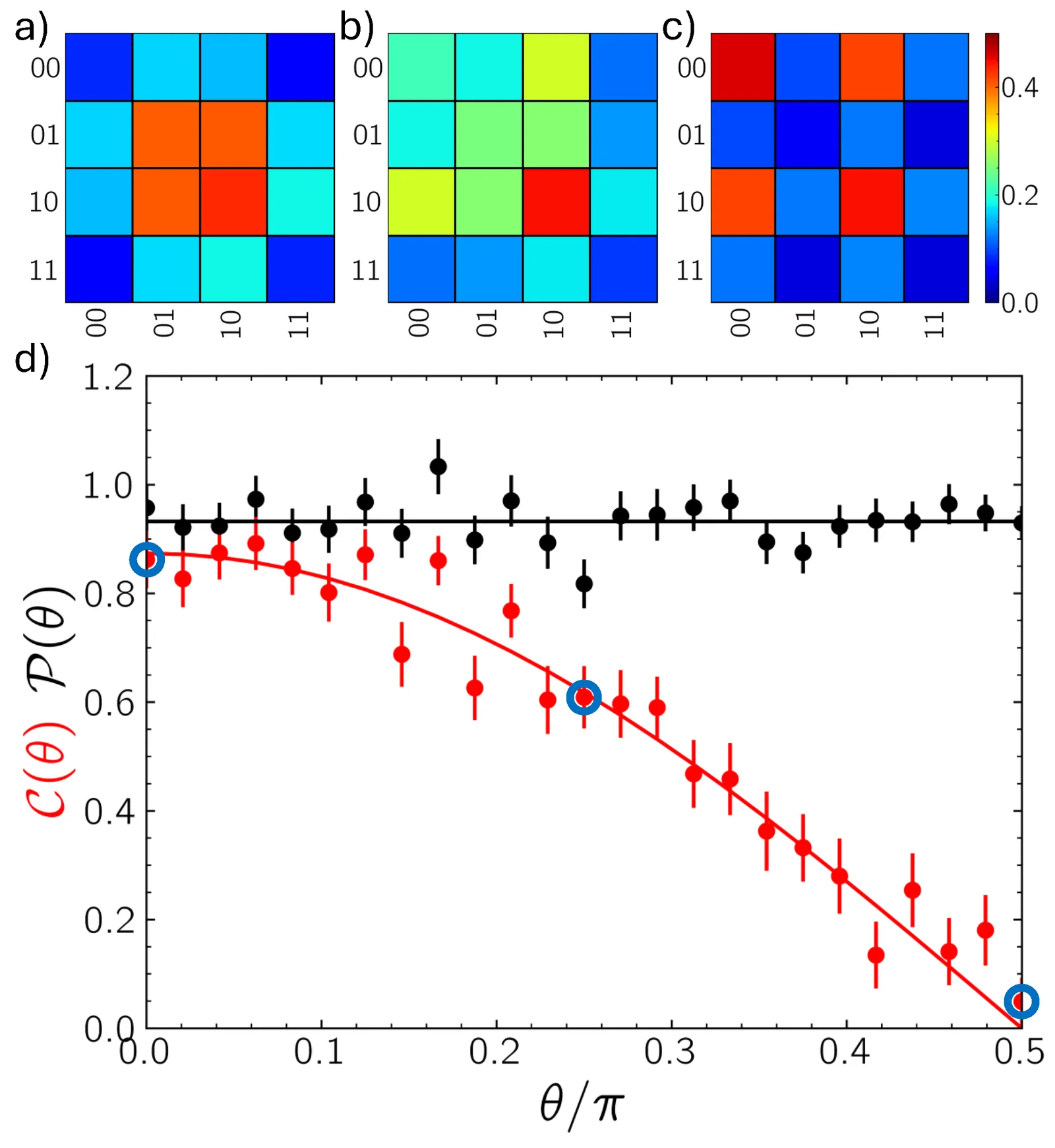
Characterization of the resource state: (**d**) Measured concurrence (red) and purity (black) versus the preparation angle θ. The red solid line is the fit $C\left(\theta \right)=C_{0}\cos\theta$, while the black solid line represents the mean purity value. For each data point, quantum state tomography with 100 shots per Pauli operator was carried out; the confidence intervals were computed via parametric bootstrapping (see the text). The top panels (**a**–**c**) show reconstructed density matrices (absolute values) for the values marked with blue circles—θ=π×{0,0.25,0.5}.

**Figure 4:**
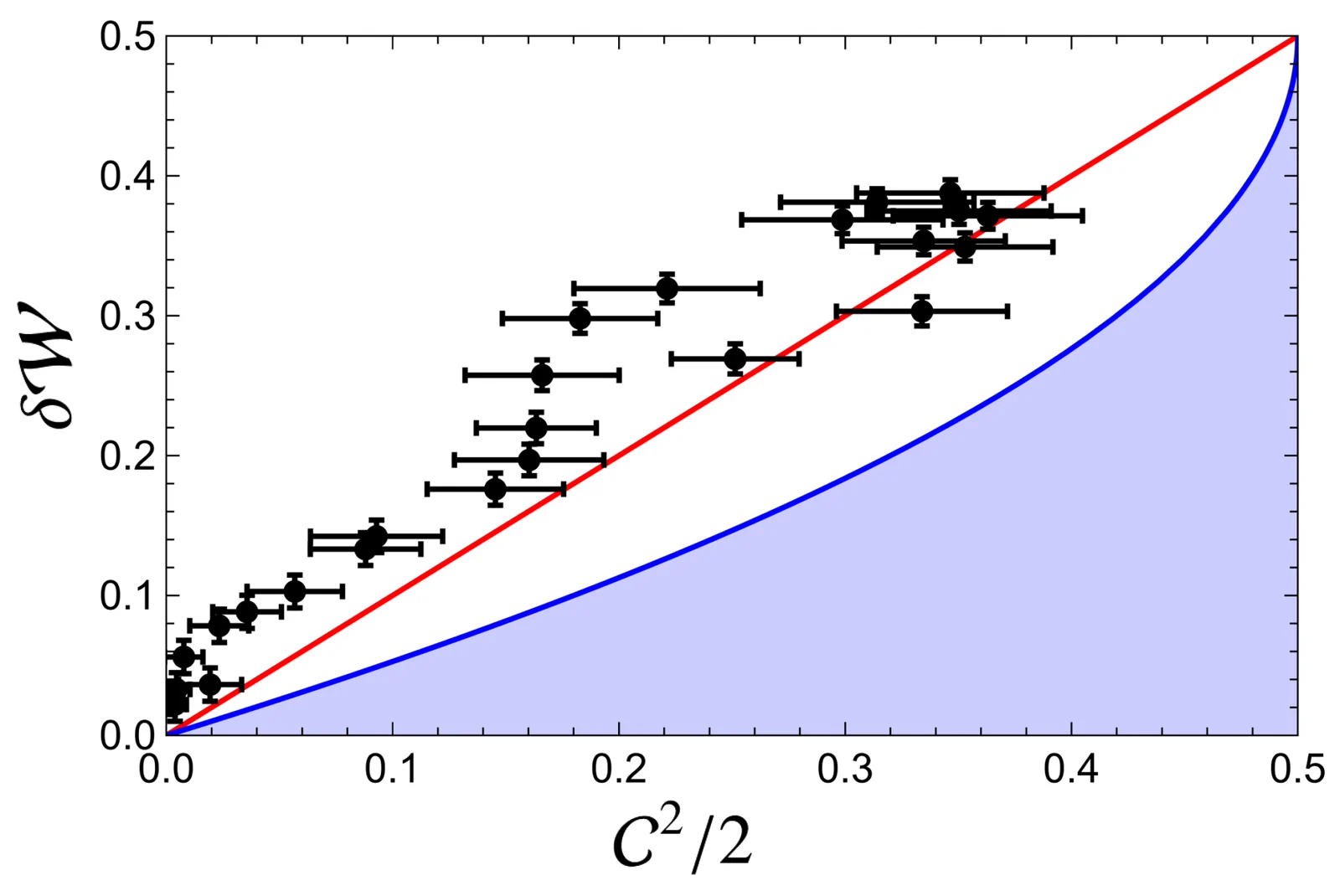
Characterization of the feedback-assisted energy gain δW: The values for δW computed from the final populations shown in Figure 2 according to Equations (10) and (11) are plotted versus $C{\left(\theta \right)}^{2}/2$, with the concurrence obtained from Figure 3. Note that the statistical error of $C^{2}$ is proportional to C. The red line is the prediction for the selected pure-state family, Equation (8); the blue line is the demonic ergotropy lower bound from Ref. [27], Equation (1), which is shown only for comparison and not as a bound derived for the fixed conditional-SWAP protocol.

A separate series of measurements was carried out to analyze the relevant properties of the resource state via quantum state tomography. The initial state Equation (2) was prepared by executing the circuit shown in Figure 1 up to and including the parametric controlled-*Y* gate for all values of θ. For each setting, one of nine Pauli gates for measuring both qubits along the respective axes was applied. For each measurement setting, 100 shots were acquired, and the resulting population estimates were used to reconstruct the density matrix $\hat{\rho }$ of the resource state via linear inversion. This allows the computation of estimates for the concurrence C(θ) via Equation (3) as well as the purity $P\left(\theta \right)=\mathrm{Tr}\left({\hat{\rho }}^{2}\right)$. The resulting values for the concurrence and purity are shown in Figure 3. Confidence intervals for all quantities were computed via parametric bootstrapping: For each value of θ, 500 artificial population data sets were computed from the reconstructed density matrix by sampling from the multinomial distribution determined by its diagonal. This data in turn is used to again compute density matrices via linear inversion, from which the metrics are computed and used to provide a measure of statistical spread. Density matrices obtained from linear inversion of finite measurement data are not necessarily positive; consequently, nonlinear quantities such as concurrence and purity inherit a reconstruction-dependent systematic uncertainty, and unphysical estimates such as P>1 can occur. We therefore regard the concurrence values as tomography-based estimates and do not infer an entanglement-only origin of the independently measured feedback gain from them. The direct feedback-gain data, by contrast, are obtained from measured frequencies without density-matrix reconstruction. The consistently high purity estimates are compatible with the intended nearly pure state family. Fitting the concurrence values to $C\left(\theta \right)=C_{0}\cos\theta$ reveals a consistent reduction factor $C_{0}=0.87\left(2\right)$, which is attributed to gate errors (see below). The measured feedback-assisted gain versus the estimated concurrence squared is shown in Figure 4. Within statistical accuracy, the energy-gain values follow the pure-state-family relation in Equation (8) up to an offset, revealing a systematic deviation that can be described by a microscopic gate-error model. We empirically model imperfections of the controlled-*Z* gates [38], taking into account that, when such gates are applied after shuttling operations, residual ion motion can lead to gate phases deviating from the preset value π/2. Simulating the entire circuit using *Qiskit* [41], we fit the measured population data and obtained gate phase deviations $\delta \Phi =\frac{\pi }{2}\times \left\{0.009\left(2\right),0.068\left(9\right),0.165\left(8\right)\right\}$ for the three controlled-*Z* gates used in the circuit. These parameters were inferred from the same data represented by the solid lines in Figure 2; they are not independent calibrations. The model should therefore be understood as a phenomenological consistency check identifying controlled-phase errors as a plausible source of the systematic deviations rather than as an independently validated prediction. As the concurrence values fall short of the ideal values by the factor $C_{0}$, the feedback-gain values shown in Figure 4 systematically exceed the pure-state-family prediction Equation (8).

## 5. Discussion and Outlook

We have experimentally realized an agent–demon protocol in which the demon employs real-time feedback on a microscopic system, conditioned on measurement outcomes, to increase the energy stored in her qubit. For the investigated family of pure input states, the measured feedback gain follows the predicted dependence on the independently estimated concurrence. We emphasize that for the state family used in this work, concurrence, the population $\mathrm{Pr}(1_{A}0_{D})$, and the resource energy exhibit the same variation with respect to the preparation parameter θ. Our results constitute an experimental test of correlation-assisted energy transfer within the chosen family, not a demonstration of an advantage unique to non-classical correlations. This distinction is consistent with the general result that classically correlated states can also yield nonzero daemonic gain [27]. The experiment opens up perspectives for controlled comparisons of classical and non-classical correlations with respect to fixed local populations or fixed total energy and for studies of non-isodimensional subsystems that explore the intermediate regime between a well-defined microscopic subsystem and a reservoir. It would also be interesting to determine feedback-assisted work using explicitly implemented work storage and to compare it with ergotropy-based definitions [15,42].

## Data Availability

The raw data supporting the conclusions of this article will be made available by the authors on request.

## Supplementary Materials

The following supporting information can be downloaded at https://www.mdpi.com/article/10.3390/e28101079/s1, Figure S1: Relevant electronic states and optical transitions of ^40^Ca+ ions, used for encoding the spin qubits employed in the work; Figure S2: Decomposition of the parametric controlled-*Y* rotation (Figure 1d) from the main manuscript.
