# Supplementary material for: Experimental Demonstration of Correlation-Assisted Energy Extraction Gain
Source: Entropy (Basel). 2026 Sep 30;28(10):1079. doi: 10.3390/e28101079 (PMC13703142; doi:10.3390/e28101079)
Supplement: Supplementary file 1 [file entropy-28-01079-s001.zip › entropy-4512362-supplementary.pdf]

Experimental Demonstration of Correlation-Assisted Energy Extraction Gain: Supplemental Material

A. Stahl,[1] M. Kewming,[2] J. Goold,[2] J. Hilder,[1] U. G. Poschinger,[1,*] and F. Schmidt-Kaler[1]

[1]*Institut für Physik, Universität Mainz, Staudingerweg 7, 55128 Mainz, Germany*

[2]*School of Physics, Trinity College Dublin, College Green, Dublin 2, Ireland*

(Dated: September 21, 2026)

I. QUBIT IMPLEMENTATION AND OPERATIONS

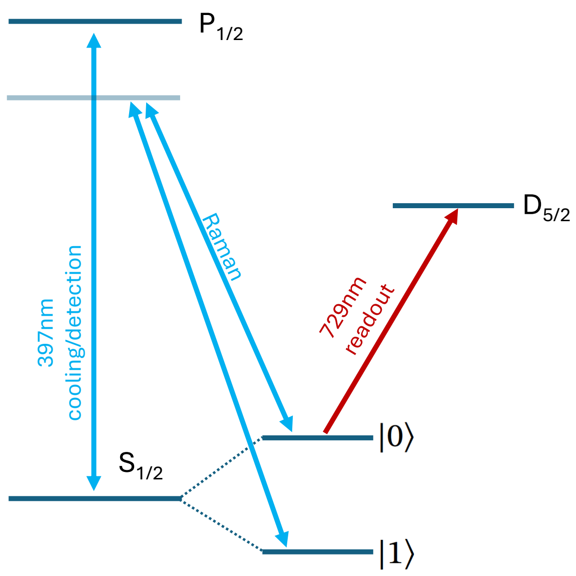

Figure S1. Relevant electronic states and optical transitions of $^{40}Ca^{+}$ ions, used for encoding the spin qubits employed in the work.

We employ qubits encoded in the spin of the valence electron of trapped $^{40}Ca^{+}$ ions, i.e. in the two Zeeman sublevels of the $S_{1/2}$ electronic ground state. The relevant part of the atomic level scheme is shown in Fig. 1. The Zeeman splitting between the two qubit energy levels, induced by an external magnetic field, is about $\epsilon/\hbar \approx 2\pi{\times}10$ MHz. Note that the all relevant quantities reported in the main manuscript are normalized to the qubit energy ϵ. The ions are trapped and cooled via illumination on the $S_{1/2} \leftrightarrow P_{1/2}$ electric dipole transition near 397 nm. The resonance fluorescence photons emitted upon exposure to the 397 nm light also serve for state-dependent detection. As the resonance fluorescence detection does not discriminate the qubit levels, an addition electron shelving step is performed upon qubit readout. Here, population is selectively transferred from state $|0\rangle$ to the metastable $D_{5/2}$ state via rapid adiabatic passage. The subsequent detection of resonance fluorescence on a photomultiplier tube then only yields a signal for qubit state $|1\rangle$. The combined error of the state discrimination is about 0.3%.

Off-resonant laser beams detuned by about 600 GHz from the $S_{1/2} \leftrightarrow P_{1/2}$ transition are used for driving quantum gate operations via stimulated Raman transitions. Different beam pair arrangements are used for different purposes. All beams are individually controlled in frequency, optical phase and intensity via single-pass acousto-optical modulators. A pair of beams, directed such that their difference wavevector is perpendicular to a two-ion Coulomb crystal, drives the qubit such that the drive is sensitive to harmonic motion along this direction. This way, motional sidebands of the qubit transition can be driven, which is used for cooling collective modes of the two-ion crystal close to the ground state via resolved sideband cooling, which is a crucial prerequisite for realizing entangling gate operations. A pair of off-resonant beams aligned perpendicular to the quantizing magnetic fields is employed for realizing local qubit rotations of the form

$$\hat{R}(\theta,\phi) = \exp\left[-\tfrac{i}{2}\theta\left(\cos\phi\hat{X} + \sin\phi\hat{Y}\right)\right] \qquad (1)$$

Here, the rotation angle θ is given by the pulse area and controlled via the duration of the pulse (typically about 10 μs for a $\theta = \pi$ rotation). The rotation phase ϕ, controlling the rotation axis on the Bloch sphere, is controlled via the optical phase difference of the two beams. Finally, a beam pair again having an effective wavevector along direction perpendicular to the ion crystal, with mutually perpendicular optical polarization, is employed for realizing entangling phase gates via spin-dependent optical dipole forces. The interference of the beams gives rise to a pattern of periodically alternating optical polarization across the ions, which in turn gives rise to a spatially alternating spin-dependent ac-Stark shift. Introducing a frequency difference close to a secular mode frequency between the two beams then gives rise to an oscillatory force on the ions, being near-resonant to the respective mode and its sign depending on the spin configuration of the two qubits. This in turn leads to a parity-dependent pickup of a geometric phase, which results in the desired entangling phase gate

$$\hat{U}_{ZZ} = \exp\left[-\frac{i}{2}\Phi\hat{Z}\otimes\hat{Z}\right] \qquad (2)$$

the gate phase $\Phi = \pi/4$ is controlled via the optical power of the beams, for a fixed drive time of 100 μs. Two such gates interspersed with a rephasing rotation $\hat{R}(\pi,0)$ then yields a maximally entangling gate. The rephasing pulse mainly serves for cancelling an unbalanced residual background ac-Stark, which would lead to spurious local qubit rotations.

II. CIRCUIT CONSTRUCTIONS

In this section, we explain the decomposition of the parametric controlled-Y gate shown in Fig. 1d) (main manuscript).

* poschin@uni-mainz.de

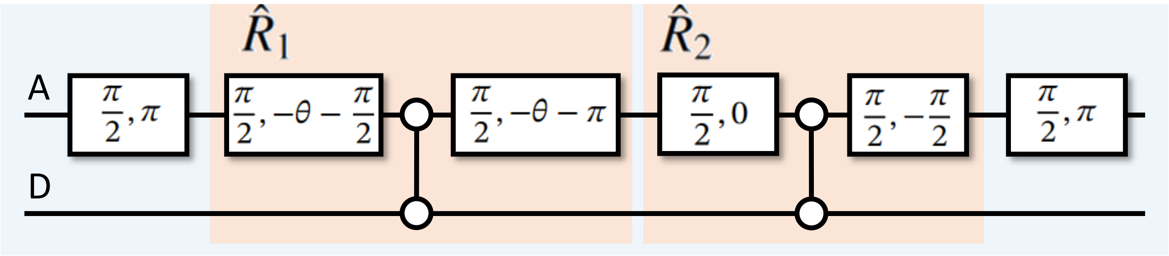

Figure S2. Decomposition of the parametric controlled-Y rotation (Figure 1 (d) from the main manuscript).

We first note that - ignoring the rephasing pulses for now - the entangling phase gate can be expressed as a controlled-Z gate ($c\hat{Z} = \mathbb{1} - 2|11\rangle\langle 11|$) and a simultaneous local Z rotation by $\pi/2$ on both qubits:

$$\hat{G} = c\hat{Z}\,\hat{R}_z^{(A,D)}(-\tfrac{\pi}{2}) \quad (3)$$

We now focus on the upper lead (qubit A in Fig. 1(d)). The local Z-rotation from the $\hat{G}$ gate changes the effective phase angle of any subsequent qubit rotation $\hat{R}(\theta,\phi)$ by $-\pi/2$:

$$\hat{R}(\theta,\phi)\hat{R}_z(-\tfrac{\pi}{2}) = \hat{R}_z(-\tfrac{\pi}{2})\hat{R}(\theta,\phi+\tfrac{\pi}{2}) \quad (4)$$

For the case that qubit D is in $|0_D\rangle$, the $c\hat{Z}$ gate has no effect, and the net effect of first gate blocks on A is

$$\begin{aligned} \hat{R}_1 &= \hat{R}\left(\tfrac{\pi}{2},-\theta-\pi\right)\hat{R}_z\left(-\tfrac{\pi}{2}\right)\hat{R}\left(\tfrac{\pi}{2},-\theta-\tfrac{\pi}{2}\right) \\ &= \hat{R}_z\left(-\tfrac{\pi}{2}\right)\hat{R}\left(\pi,-\theta-\tfrac{\pi}{2}\right) \\ &= i\hat{R}_z\left(-\tfrac{\pi}{2}\right)\left(\cos\theta\hat{Y}+\sin\theta\hat{X}\right) \quad (5) \end{aligned}$$

Similarly, the second gate block has the net effect on A:

$$\begin{aligned} \hat{R}_2 &= \hat{R}\left(\tfrac{\pi}{2},-\tfrac{\pi}{2}\right)\hat{R}_z\left(-\tfrac{\pi}{2}\right)\hat{R}\left(\tfrac{\pi}{2},0\right) \\ &= \hat{R}_z\left(-\tfrac{\pi}{2}\right)\hat{R}\left(\pi,0\right) \\ &= -i\hat{R}_z\left(-\tfrac{\pi}{2}\right)\hat{X} \quad (6) \end{aligned}$$

The combined net effect of both blocks is thus

$$\hat{R}_2\hat{R}_1 = i\cos\theta\hat{Z} + \sin\theta\,\mathbb{1} \quad (7)$$

Together with the outer $\pi/2$ rotations, this finally gives

$$\begin{aligned} \hat{R}\left(\tfrac{\pi}{2},\pi\right)\hat{R}_2\hat{R}_1\hat{R}\left(\tfrac{\pi}{2},\pi\right) &= i\cos\theta\hat{Z} + i\sin\theta\hat{X} \\ &= i\hat{Z}\left(\cos\theta\,\mathbb{1} + i\sin\theta\,\hat{Y}\right) \\ &= i\hat{Z}\hat{R}_y(\theta) \quad (8) \end{aligned}$$

For the case that qubit D is in $|1_D\rangle$, according to Eq. 3 the $c\hat{Z}$ gate effects an additional $\hat{Z}$ gate on A. Using that $\hat{R}_z(-\pi/2)\hat{Z} = i\hat{R}_z(\pi/2)$, the net effect of the gate block 1 on A is then

$$\begin{aligned} \hat{R}_1 &= i\hat{R}\left(\tfrac{\pi}{2},-\theta-\pi\right)\hat{R}_z\left(\tfrac{\pi}{2}\right)\hat{R}\left(\tfrac{\pi}{2},-\theta-\tfrac{\pi}{2}\right) \\ &= i\hat{R}_z\left(\tfrac{\pi}{2}\right)\hat{R}\left(\tfrac{\pi}{2},-\theta-\tfrac{3\pi}{2}\right)\hat{R}\left(\tfrac{\pi}{2},-\theta-\tfrac{\pi}{2}\right) \\ &= i\hat{R}_z\left(\tfrac{\pi}{2}\right) \quad (9) \end{aligned}$$

For second gate block, we get:

$$\begin{aligned} \hat{R}_2 &= i\hat{R}\left(\tfrac{\pi}{2},-\tfrac{\pi}{2}\right)\hat{R}_z\left(\tfrac{\pi}{2}\right)\hat{R}\left(\tfrac{\pi}{2},0\right) \\ &= i\hat{R}_z\left(\tfrac{\pi}{2}\right)\hat{R}\left(\tfrac{\pi}{2},-\pi\right)\hat{R}\left(\tfrac{\pi}{2},0\right) \\ &= i\hat{R}_z\left(\tfrac{\pi}{2}\right) \quad (10) \end{aligned}$$

and for the combined effect:

$$\hat{R}_2\hat{R}_1 = -i\hat{Z} \quad (11)$$

The effect with the $\pi/2$ rotations is then:

$$\hat{R}\left(\tfrac{\pi}{2},\pi\right)\hat{R}_2\hat{R}_1\hat{R}\left(\tfrac{\pi}{2},\pi\right) = -i\hat{Z} \quad (12)$$

Putting together Eqs. 7 and 12, we observe that the combined net effect is indeed a control-Y gate on qubit A, up to a $\hat{Z}$ and a $c\hat{Z}$ gate, both of which are irrelevant as the controlled-Y gate is followed by phase-insensitive operations.

III. DEMONIC GAIN FROM CONCURRENCE

In this section, we derive that Eq. 8 results from evaluating Eq. 7 (main manuscript) for the specific protocol. The density operators describing the initial state Eq. 2 reads:

$$\begin{aligned} \hat{\rho} = & \tfrac{1}{2}\left(\cos^2\theta\,|1_A0_D\rangle\langle 1_A0_D| + \sin^2\theta\,|0_A0_D\rangle\langle 0_A0_D|\right. \\ &+ |0_A1_D\rangle\langle 0_A1_D| \\ &+ \cos\theta\,|1_A0_D\rangle\langle 0_A1_D| + \cos\theta\,|0_A1_D\rangle\langle 1_A0_D| \\ &- \sin\theta\,|0_A0_D\rangle\langle 0_A1_D| - \sin\theta\,|0_A1_D\rangle\langle 0_A0_D| \\ &- \left.\cos\theta\sin\theta\,|1_A0_D\rangle\langle 0_A0_D| - \cos\theta\sin\theta\,|0_A0_D\rangle\langle 1_A0_D|\right) \end{aligned}$$

The initial energy locally available to the demon, in units of the qubit energy is:

$$E_i/\epsilon = \mathrm{Tr}_{AD}\left(\hat{H}_D\hat{\rho}\right) = \frac{1}{2} \quad (13)$$

The post-measurement states result from tracing out the demon's qubit, conditioned on the demon's measurement result:

$$\begin{aligned} \hat{\rho}_{0_D} &= \langle 0_D|\hat{\rho}|0_D\rangle \otimes |0_D\rangle\langle 0_D| \\ &= \left(\cos^2\theta\,|1_A\rangle\langle 1_A| + \sin^2\theta\,|0_A\rangle\langle 0_A|\right. \\ &\quad - \left.\cos\theta\sin\theta\,|1_A\rangle\langle 0_A| - \cos\theta\sin\theta\,|0_A\rangle\langle 1_A|\right) \otimes |0_D\rangle\langle 0_D| \\ \hat{\rho}_{1_D} &= \langle 1_D|\hat{\rho}|1_D\rangle \otimes |1_D\rangle\langle 1_D| \\ &= |0_A\rangle\langle 0_A| \otimes |1_D\rangle\langle 1_D| \quad (14) \end{aligned}$$

The state $\hat{\rho}'$ after conditional SWAP (Eq. 6 from the main manuscript) completely written out is obtained from swapping $0_D \leftrightarrow 1_A$ for $\hat{\rho}_{0_D}$ and adding the unchanged density operator $\hat{\rho}_{1_D}$:

$$\begin{aligned} \hat{\rho}' = & \tfrac{1}{2}\left(\cos^2\theta\,|1_D\rangle\langle 1_D| + \sin^2\theta\,|0_D\rangle\langle 0_D|\right. \\ &- \cos\theta\sin\theta\,|1_D\rangle\langle 0_D| - \cos\theta\sin\theta\,|0_D\rangle\langle 1_D| \\ &+ \left.|1_D\rangle\langle 1_D|\right) \otimes |0_A\rangle\langle 0_A| \quad (15) \end{aligned}$$

The agent thus always ends up in $|0_A\rangle$:

$$\hat{\rho}'_A = \mathrm{Tr}_D\hat{\rho}' = |0_A\rangle\langle 0_A| \tag{16}$$

The final state as locally seen by the demon is:

$$\begin{aligned}\hat{\rho}'_D &= \mathrm{Tr}_A\hat{\rho}' \\ &= \tfrac{1}{2}\Big((1+\cos^2\theta)\,|1_D\rangle\langle 1_D| + \sin^2\theta\,|0_D\rangle\langle 0_D| \\ &- \cos\theta\sin\theta\,(|1_D\rangle\langle 0_D| + |0_D\rangle\langle 1_D|)\Big)\end{aligned} \tag{17}$$

Therefore, the final energy locally available to the demon is

$$E_f/\epsilon = \mathrm{Tr}_D\left(\hat{H}_D\hat{\rho}'_D\right) = \frac{1}{2}(1+\cos^2\theta) \tag{18}$$

We finally obtain the demonic gain:

$$\frac{1}{\epsilon}(E_f - E_i) = \frac{\cos^2\theta}{2} = \frac{C(\theta)^2}{2} \tag{19}$$

which shows that the general theory result (Eq. 8 from the main manuscript) holds for our specific instance of an agent-demon energy extraction protocol.

IV. CONCURRENCE MEASUREMENT

For the characterization of the resource state, we perform measurements of nine different two-qubit Pauli operators $XX, XY, XZ, YX, YY, YZ, ZX, ZY, ZZ$ for each preparation angle θ. For a Pauli X measurement on any of the two qubits, a Hadamard gate is executed prior to readout in the logical (Z) basis, while for Pauli Y measurements, a $\pi/2$ phase gate and subsequent Hadamard gate are executed before readout. For each qubit readout, we assign the results 'dark' $\rightarrow +1$ and 'bright' $\rightarrow -1$ and multiply the results for both qubits. The results for each two-qubit Pauli operator is averages over 100 shots. The resulting nine estimated populations are fed into a linear inversion routine of Qiskit to obtain a reconstruction of the two-qubit density matrix.

Estimates for the statistical errors for the concurrence and purity values are obtained from parametric bootstrapping. Here, we take the measured populations $\mathrm{Pr}(M(\hat{P}) = +1)$ for each Pauli operator $\hat{P}$ to be the parameters underlying a binomial distribution for $N = 100$ shots. Then, for each reconstructed density matrix, we generate 500 random instances of artificial measurement data by drawing the nine populations from a binomial distribution $\mathrm{Binom}(N, p)$. For each random instance, we reconstruct a density matrix and evaluate its purity and concurrence. The error bars for these shown in main manuscript result are given by one standard deviation of the artificially generated values.

The horizontal error bars shown in Fig. 4 of the main manuscript considerably vary in size. The reason for this is that these pertain to the concurrence squared, for which the statistical errors is given by $C\sigma_S$, i.e. it scales with the mean concurrence.
